# Efficacy of mitomycin-C on anterior urethral stricture after internal urethrotomy: A systematic review and meta-analysis

**DOI:** 10.12688/f1000research.19704.1

**Published:** 2019-08-08

**Authors:** Gampo Alam Irdam, Irfan Wahyudi, Andy Andy

**Affiliations:** 1 Department of Urology, Faculty of Medicine, Universitas Indonesia, Jakarta Pusat, DKI Jakarta, 10430, Indonesia

**Keywords:** mitomycin-c, urethral stricture, internal urethrotomy

## Abstract

**Background: **Mitomycin-C is an agent that plays an important role in the tissue healing process and scar formation. This study aims to investigate the efficacy of mitomycin-C in treating anterior urethral stricture following internal urethrotomy.

**Methods: **Studies evaluating the efficacy of mitomycin-c for anterior urethral stricture post urethrotomy were searched using PubMed, Scopus, ScienceDirect, EBSCOHost, Cochrane Reviews, and OVID as directory databases. Terms used in the searching process were “mitomycin-c” or “mitomycin”, “urethral stricture”, “urethral stenosis”, “internal urethrotomy”, “optical urethrotomy” and its synonyms. Every randomized controlled trial study conducted in human subjects was included. Study appraisals were conducted in accordance with Oxford University Center for Evidence-Based Medicine guidelines. The conclusion of each study was summarized and the calculation of fixed effect from every study was conducted in meta-analysis.

**Results: **Included in this study were three studies involving 231 patients. All of them reported less recurrence of in patients treated with mitomycin-c post urethrotomy (p<0.001). The fixed risk ratio of all studies was 0.32 with 95% confidence interval (0.19-0.54). All studies also reported less stricture length after treatment with mitomycin-c, but there were not statistical differences between with or without treatment group.

**Conclusion: **Mitomycin-C has efficacy in treating anterior urethral stricture after internal urethrotomy. However, the inclusion of relatively few studies may affect the strength of this review and further studies need to be done.

## Background

Urethral stricture often impairs quality of life and may result in a large economic burden ^1^. There are several procedures available for treating this condition, ranging from minimally invasive procedures like internal optical urethrotomy (IOU) to invasive procedure such as urethroplasty, with or without grafting, and tissue engineering ^2^. However, despite the methods available, urethral stricture often recurs. Several manipulations have been tried to prevent urethral stricture, such as indwelling catheter insertion, urethral calibration procedure, and home self-catheterization. Unfortunately, repeated instrumentation can cause scar formation. Moreover, it can also complicate subsequent reconstruction, which can lead to several complications ^3, 4^. On the other hand, there have been several studies evaluating the effects of antifibrotic drugs such as glucocorticoid and mitomycin-C on urethral strictures. Mitomycin-C is an agent that has the potential to inhibit mitosis, fibroblast proliferation, formation of blood vessels, and synthesis of protein and collagen. This agent plays role in tissue healing process and scar formation by reducing the release of matrix proteins by inhibiting proliferative fibroblasts ^5^.

To our knowledge, there have not been any systematic reviews or meta-analyses regarding the efficacy of mitomycin-C in treating anterior urethral stricture post internal urethrotomy. Thus, the present study aims to investigate the efficacy of mitomycin-C in treating anterior urethral stricture post internal urethrotomy. We hope that by conducting this review and analysis, a definite conclusion regarding the efficacy of such treatment could be achieved.

## Methods

This systematic review was conducted based on guidelines from the Oxford University Center for Evidence-Based Medicine ^6^. Our present study aims to determine whether mitomycin-C provide better efficacy compared to controls (without mitomycin-C) in adult patients with anterior urethral stricture after internal urethrotomy.

### Inclusion and exclusion criteria

To be considered for inclusion, the included studies were required to be randomized controlled trials (RCTs) study investigating efficacy of mitomycin-C as the additional treatment to internal urethrotomy in anterior urethral stricture. We expanded the searching by including related studies suggested by the databases. Year of publishing was not considered as inclusion criterion. Any study until September 22th 2018 was included. The primary outcome measures were efficacy of mitomycin-C administration, determined by risk ratio for proportion results and mean difference for continuous data. Animal studies, case series, case report, editorials, and book chapters were excluded.

### Search strategy

To find suitable studies to be included in this review, we used PubMed, Scopus, ScienceDirect, EBSCOHost, Cochrane Reviews, and OVID as directory databases. We used combination of keywords “((((((mitomycin c[MeSH Terms]) OR mitomycin[MeSH Terms]) OR mitomycin c)) AND ((((((((((((((((urethral stricture[MeSH Terms]) OR urethral strictures[MeSH Terms]) OR stricture, urethral[MeSH Terms]) OR strictures, urethral[MeSH Terms]) OR urethral stenosis[MeSH Terms]) OR urethral stenoses[MeSH Terms]) OR stenosis, urethral[MeSH Terms]) OR stenoses, urethral[MeSH Terms]) OR urethral stricture) OR urethral strictures) OR stricture, urethral) OR strictures, urethral) OR urethral stenosis) OR urethral stenoses) OR stenosis, urethral) OR stenoses, urethral)) AND “urethra/surgery”[MeSH Terms]) AND Humans[Mesh]”. We also used term “human” as limiting term to exclude every study that was not conducted on human subjects.

### Analysis and concluding the review

We evaluated the study using appraisal worksheet for randomized clinical trial from Oxford University Center for Evidence-Based Medicine to stratify the risk of bias ^6^. Using Revman 5.3 software, inputted data of stricture length (in mm) and recurrence number from all selected studies were analyzed. Data were analyzed using the homogeneity index (I ^2^) and forest plots. Calculation of fixed effect was also done using Revman 5.3 to show relative risk/risk ratio for recurrence rate variable, mean difference for stricture length, dan p-value for both variables. We summarized the conclusion of each study at the table along with its appraisal.

## Results

### Literature search

**Figure 1.**
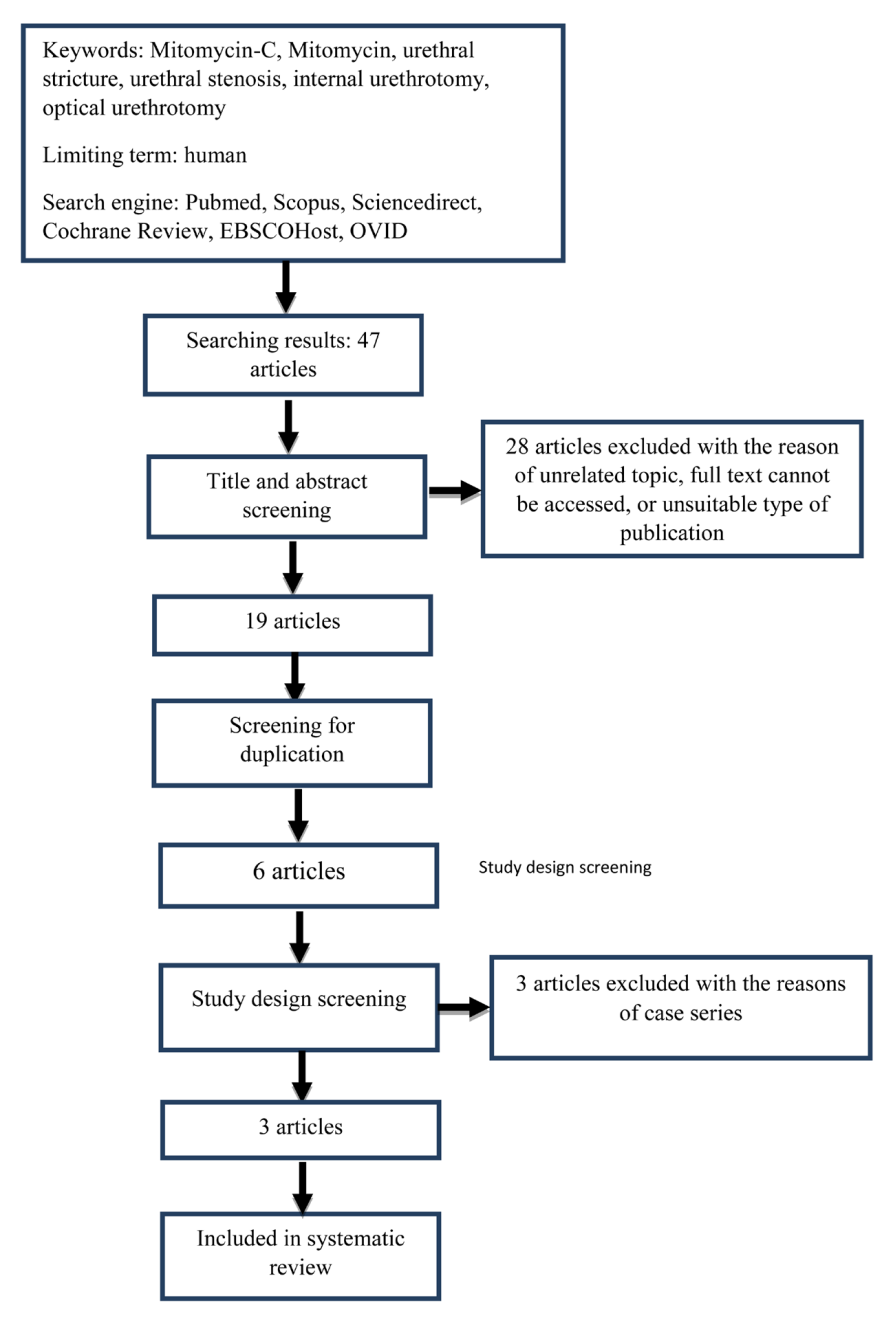
Study flow diagram.

Searching process (searching strategy showed in Figure 1) by using six databases found 47 study articles. There were 28 articles eliminated after title and abstract screening. The remained 19 articles were reduced to six articles after eliminating duplicates, leaving six full text articles to be reviewed. Based on study design, we eliminated three articles, leaving three articles to be summarized in systematic review and meta-analysis.

### Study characteristics and quality assessment

Three selected studies were conducted in 2007, 2015, and 2016 ^2, 4, 7^. All studies evaluated the effectivity of mitomycin-C given after internal urethrotomy for anterior urethral stricture. From these selected articles, two evaluated the usage of submucosal injection of mitomycin-C for anterior urethral stricture after urethrotomy. How mitomycin-C was injected differed in both studies; one study used 0.1 mg mitomycin-C in 2 ml distilled water injected in four quadrants while another study used 0.1% mitomycin-C injected into three quadrants. One study evaluated the intraluminal injection of mitomycin-C in a hydrogel base. It consists of 0.8 mg mitomycin-C with 1 cm ^3^ propylene glycol and water to PF-127 poloxamer. The hydrogel base was injected through a small feeding tube to reach the site of stricture. All studies applied mitomycin-C after internal urethrotomy procedure and were conducted in populations with different age means. Each of studies’ quality was assessed using guide from Oxford University Center for Evidence-Based Medicine; this is explained in Table 1.

**Table 1.** Characteristics of subjects in included studies.

Studies	Average age (year)		Pre-intervention stricture site		Procedure of internal urethrotomy	Clinical feature				Cause of injury	
	MMC group	Control group	MMC Group	Control group		Urinary retention	Bladder Outlet Obstruction	Azotemia	Urosepsis	Mitomycin-C Group	Control group
Moradi *et al*., 2016 ^7^	54.55 ± 21.25	53.75 ± 24.75	Anterior urethral stricture		Trans- urethral incision at 12 o’clock via cold knife urethrotomy	N/A				N/A	
Ali *et al*., 2015 ^2^	37.31 ± 10.1	40.1 ± 11.4	Bulbar urethra: 84.6% Penile urethra: 15.4%	Bulbar urethra: 78.1% Penile urethra: 21.9%	Internal Optic urethrotomy	Mitomycin-C group				Road traffic: 59% Iatrogenic: 30.8% Straddle: 9% Infective: 1.3%	Road traffic: 71.2% Iatrogenic: 21.9% Straddle: 6.8% Infective: -
						70.5%	16.6%	10.2%	2.5%		
						Control group					
						57.5%	31.5%	9.5%	1.3%		
Mazdak *et al*., 2007 ^4^	29.8 (15-70)	29.2 (11-66)	Anterior urethral stricture		Trans- urethral incision at 12 o’clock via cold knife urethrotomy	N/A				Straddle injury or other blunt perineal trauma	

MMC, mitomycin C.

**Table 2.** Summary and appraisal of the selected articles.

Studies	LoE	Sample size	Methods of mitomycin-C application	Timing of mitomycin-C application	Follow up end-point	Validity					Importance	Applicability		
						Randomized allocation	Similarity of group	Equal Treatment	Minimal Loss to Follow-up	Blinding		Relevance	Feasibility	Benefit Overweight the Harm
Moradi *et al.*, 2016 ^7^	1b	40	Intraluminal injection of 0.8 mg mitomycin-C + propylene glycol through indwelling catheter	After Internal Urethrotomy	12 months	Yes	Yes	Yes	Yes	Not stated	RR = 0.20 ARR = 0.40 RRR = 0.80 NNT = 1	Unsure	Yes	Yes
Ali *et al.*, 2015 ^2^	1b	180	Submucosal injection of 0.1% mitomycin-C at three quadrants (1, 11, & 12 o’clock position) using TLA needle	After Internal Urethrotomy	18 months	Yes	No	Yes	Yes	Not stated	RR = 0.38 ARR = 0.23 RRR = 0.62 NNT = 2	Unsure	Yes	Yes
Mazdak *et al.*, 2007 ^4^	1b	40	Submucosal injection of 0.1 mg mitomycin-C in four quadrants (1,5,7, & 11 o’clock position) using 22- Gauze cystoscopic needle	Before Internal Urethrotomy	6 months	Not stated	Yes	Yes	Yes	Not stated	RR = 0.20 ARR = 0.40 RRR = 0.80 NNT = 1	Unsure	Yes	Yes

LoE, level of evidence; RR relative risk; ARR, absolute risk reduction; RRR, relative risk reduction; NNT, number needed to treat.

### Outcome measures

We included the studies in which stricture was measured using retrograde urethrogram or ultrasonography of the urethra. The outcome that measured was recurrence rate (percentage) and stricture length (mm) after treatment.

### Results and heterogeneity of the studies

**Figure 2.**
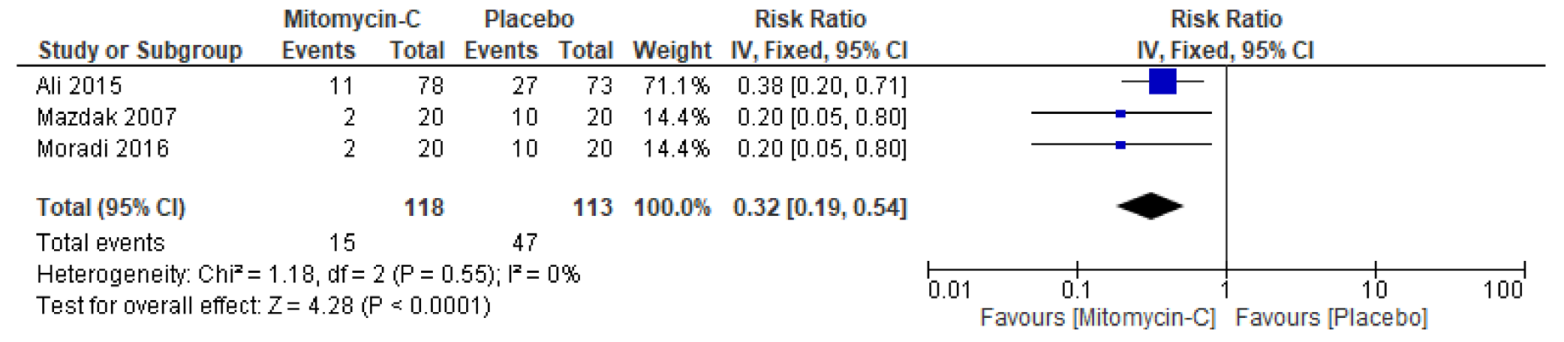
Forest plot for recurrence rate. Test for heterogeneity chi-square = 1.18; df(Q) = 2; p-value < 0.0001; I ^2^ < 0.001.

All selected articles stated that mitomycin-C had a significant effect on preventing or delaying urethral stricture recurrence post internal urethrotomy. All studies reported that the time-based recurrence rates in the two groups differed, where lower recurrence rates were found in the group given mitomycin-C ^2, 4, 7^. The forest plot in Figure 2 showed that recurrence rate are homogenous in all analyzed studies (I ^2^ <0.001, p=0.55). This was performed by using fixed effect model in the forest plot. This forest plot suggests that there were significant differences between cases and control group. The mean recurrence rate was 0.32 (95%CI=0.19 to 0.54).

**Figure 3.**
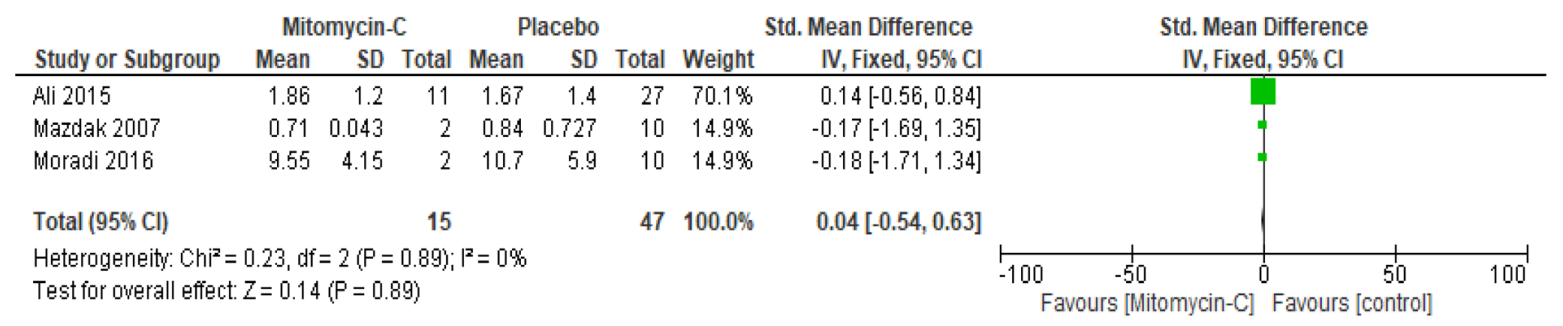
Forest plot for stricture length after treatment. Test for heterogeneity chi-square = 0.23; df(Q) = 2; p-value = 0.89; I ^2^ < 0.001.

 On the other hand, in stricture length, all studies showed that there were not significant differences between the groups that received and did not receive mitomycin-C administration (p=0.38). Figure 3 showed homogenous studies in stricture length (I ^2^ < 0,001; p=0.38) analysis. The mean difference was -0.02 (95%CI=-0.27 to 0.23).

## Discussion

Urethral stricture is a serious complication in male patients, which causes great morbidity and considerable health-related costs. This condition often results in voiding dysfunction, which will affect quality of life. Moreover, this voiding disfunction can trigger chain of events leading to renal failure. Majority of the recommended treatment methods have low success rate ^8^. A recent survey revealed that 86% of American urologists prefer internal urethrotomy when treating anterior urethral stricture ^9^. However, internal urethrotomy is associated with a high rate of urethral stricture recurrence, ranging from 20 to 60% ^10, 11^. Although several methods have been introduced to lower recurrence rate, including clean intermittent self-catheterization ^12^, numerous authors has labeled this a failure as it could not fully prevent recurrence of urethral stricture.

Despite unclear pathophysiology of urethral stricture, a pathophysiological mechanism suggested is fibrosis caused by excessive synthesis of collagen and altered extracellular matrix ^13, 14^. Therefore, any drug or procedure which can delay fibrosis after internal urethrotomy would lead to an increase of surgical success rates and patient comfort, and therefore decrease treatment costs. Therefore, many studies have been performed to explore different molecules the can prevent fibrosis and urethral stricture recurrence ^4, 13– 16^.

Mitomycin-C is an alkylating antineoplastic antibiotic, produced by *Streptomyces caespitosus*. Mitomycin-C can inhibit DNA synthesis by linking adenine and guanine, resulting in DNA cross-linking. It can suppress cellular RNA and protein synthesis, and is not cell cycle specific ^8^. Therefore, it can delay the healing process by preventing replication of fibroblasts and epithelial cells, as well as inhibiting collagen synthesis ^6, 14^. It has been shown that mitomycin-C can improve the success rates of trabeculotomy and myringotomy by preventing proliferation of fibroblasts and development of fibrosis ^18, 19^.

The anti-fibroblast activity mechanism of mitomycin-C is unknown. Experts have suggested that the reduction of fibroblast activity may be mediated by myofibroblasts apoptosis ^2, 4, 11^. As the wound closes, apoptosis of myofibroblast and vascular cells increases, indicating that this is the mechanism by which granulation tissue will lead to scarring ^20, 21^.

All the studies included in this review treated the two groups equally and had relatively small loss-to-follow-up rates. A common problem with all the studies included in this review is that there was no clear blinding statement. In the study by Mazdak *et al.* ^4^, it was not stated whether there was a randomization process in the study method. On the other hand, although Ali *et al*. ^2^ had randomized its subjects, age characteristics in the two groups were significantly different.

All studies support the use of mitomycin-C to prevent or delay anterior urethral stricture after internal urethrotomy. This was confirmed by a lower rate of recurrence rate in patients treated with mitomycin-C patients ^2, 4, 7^; we found that those who had mitomycin-C administered had lower incidence of recurrence during one year and 18 months of follow up (RR = 0.32, P < 0.001). This was also confirmed by a series of cases by Farrell *et al.* ^22^, Farrell *et al.* ^23^, and Sourial *et al*. ^24^ Mazdak *et al*. ^11^ injected mitomycin-C into the submucosal layer of the urethra and reported lower rates of stricture recurrence in patients with mitomycin-C injection. On the other hand, some researchers proposed that submucosal injection might lead to an increase in complication rates and a decrease in the duration of the effective dose within the tissue ^19^. Ayyildiz *et al.* ^25^ assessed the efficacy of mitomycin-C in preventing urethral scar by means of applying the agent topically to the traumatized region in rats. They concluded that mitomycin-C applied locally reduced fibrosis significantly in a dose-independent manner.

In stricture length, we found no statistically difference between the mitomycin-C-treated and control group in all studies. However, Mazdak *et al*. ^4^ and Moradi *et al*. ^7^, even statistically showed no significant differences between these groups, the mitomycin-C-treated group showed a decrease in stricture length. The results of the study by Mazdak *et al*. ^4^ were 0.76 mm (range:0.5-1 mm) in the mitomycin-C group compared to 0.84 mm (range:0.5-1 mm) in the control group after the procedure. Moradi *et al*. ^7^ observed stricture lengths of 10.7±5.9 and 9.55±4.15 mm for the control and mitomycin-C groups, respectively.

Although all studies support the use of mitomycin-C to prevent or delay post-urethrotomy urethral stricture, the results of this review need to be followed up with caution. The limitation of this study can be seen from only a few studies that discuss this topic. Some of the existing studies are not enough to be applied to a wider population, given that selected studies were carried out only in Iran and Pakistan (and thus may not be representative of different ethnic groups) ^2, 4, 7^. Therefore, although the side effects reported in the studies reviewed are minimal, their application needs to be carried out wisely and cautiously. Research related to this in the future can still be done with different populations.

Due to short period of follow up time in all studies, some authors ^2, 4^ concluded that the study of the use of mitomycin-C in this case needed firm results regarding long-term success. Mazdak *et al*. ^4^ added that stricture may recur within two years after internal urethrotomy. 

## Conclusion

Mitomycin-C could be used as a potential additional treatment for anterior urethral strictures after internal urethrotomy. However, further studies are required to investigate the safety and efficacy of this method for treating anterior urethral strictures, as only a limited number of studies presently exist.

## Data availability

### Underlying data

All data underlying the results are available as part of the article and no additional source data are required.

### Reporting guidelines

Open Science Framework: PRISMA checklist for ‘Efficacy of mitomycin-C on anterior urethral stricture after internal urethrotomy: A systematic review and meta-analysis’. https://doi.org/10.17605/OSF.IO/APU9B ^26^.

The updated PRISMA checklist is available under the terms of the Creative Commons Zero "No rights reserved" data waiver (CC0 1.0 Public domain dedication).
