# Efficacy of mitomycin-C on anterior urethral stricture after internal urethrotomy: A systematic review and meta-analysis

**DOI:** 10.12688/f1000research.19704.3

**Published:** 2020-08-10

**Authors:** Gampo Alam Irdam, Irfan Wahyudi, Andy Andy

**Affiliations:** 1 Department of Urology, Faculty of Medicine, Universitas Indonesia, Jakarta Pusat, DKI Jakarta, 10430, Indonesia

**Keywords:** mitomycin-c, urethral stricture, internal urethrotomy

## Abstract

*Background and Aim*

Mitomycin-C is a potent agent that plays an important role in tissue healing and scar formation. This study aims to investigate the efficacy of Mitomycin-C in treating anterior urethral stricture after internal urethrotomy.

*Methods*

Studies evaluating efficacy of mitomycin-c for anterior urethral stricture post urethrotomy were searched using Pubmed, Scopus, Sciencedirect, MEDLINE, and Cochrane Reviews as directory databases. The search was done in March 15th 2020. Terms being used in the searching process were “mitomycin-c” or “mitomycin”, “urethral stricture”, “urethral stenosis”, “internal urethrotomy”, “optical urethrotomy” and its synonyms. Every study with the design of retrospective or prospective clinical study being done in human subject was included. Study appraisal conducted in accordance to Oxford University Center for Evidence-Based Medicine. The conclusion of each study was summarized and the calculation of random effects from every study was conducted in meta-analysis. Random effects model is chosen because small number of studies and quite different.

*Results*

Three studies involving 311patients were included in this review, all of them reported less recurrence of in patients treated with mitomycin-c post urethrotomy (p<0.001). Risk ratio of all studies was 0.41 with 95% confidence interval (0.25-0.68).

*Conclusion*

Mitomycin-C has the potential of efficacy in treating anterior urethral stricture post internal urethrotomy. Relatively few numbers of studies may impact in the strength of this review and further studies need to be done.

## Background

Urethral stricture often impairs quality of life and may result in a large economic burden ^1^. There are several procedures available for treating this condition, ranging from minimally invasive procedures like internal optical urethrotomy (IOU) to invasive procedure such as urethroplasty, with or without grafting, and tissue engineering ^2^. However, despite the methods available, urethral stricture often recurs. Several manipulations have been tried to prevent urethral stricture, such as indwelling catheter insertion, urethral calibration procedure, and home self-catheterization. Unfortunately, repeated instrumentation can cause scar formation. Moreover, it can also complicate subsequent reconstruction, which can lead to several complications ^3, 4^. On the other hand, there have been several studies evaluating the effects of antifibrotic drugs such as glucocorticoid and mitomycin-C on urethral strictures. Mitomycin-C is an agent that has the potential to inhibit mitosis, fibroblast proliferation, formation of blood vessels, and synthesis of protein and collagen. This agent plays role in tissue healing process and scar formation by reducing the release of matrix proteins by inhibiting proliferative fibroblasts ^5^.

To our knowledge, there have not been any systematic reviews or meta-analyses regarding the efficacy of mitomycin-C in treating anterior urethral stricture post internal urethrotomy. Thus, the present study aims to investigate the efficacy of mitomycin-C in treating anterior urethral stricture post internal urethrotomy. We hope that by conducting this review and analysis, a definite conclusion regarding the efficacy of such treatment could be achieved.

## Methods

This systematic review was conducted based on guidelines from the Oxford University Center for Evidence-Based Medicine ^6^. Our present study aims to determine whether mitomycin-C provide better efficacy compared to controls (without mitomycin-C) in adult patients with anterior urethral stricture after internal urethrotomy.

### Inclusion and exclusion criteria

Inclusion criteria were (1) Study type: RCT (Randomized Control Trial) until March 15th 2020; (2) Intervention and comparator: internal urethrotomy with addition Mitomycin-C vs internal urethrotomy without Mitomycin-C; (3) Patients: humans with anterior urethral stricture; (4) Primary outcome: efficacy of Mitomycin-C administration, determined by risk ratio; and (5) Secondary outcome: side effects from Mitomycin-C administration. Exclusion criteria were (1) Animal study; (2) case reports); (3) case series; (4) book chapters; and (5) editorials.

### Search strategy

To find suitable studies to be included in this review, we used PubMed, Scopus, ScienceDirect, MEDLINE, and Cochrane Reviews as directory databases. We used combination of keywords “((((((mitomycin c[MeSH Terms]) OR mitomycin[MeSH Terms]) OR mitomycin c)) AND ((((((((((((((((urethral stricture[MeSH Terms]) OR urethral strictures[MeSH Terms]) OR stricture, urethral[MeSH Terms]) OR strictures, urethral[MeSH Terms]) OR urethral stenosis[MeSH Terms]) OR urethral stenoses[MeSH Terms]) OR stenosis, urethral[MeSH Terms]) OR stenoses, urethral[MeSH Terms]) OR urethral stricture) OR urethral strictures) OR stricture, urethral) OR strictures, urethral) OR urethral stenosis) OR urethral stenoses) OR stenosis, urethral) OR stenoses, urethral)) AND “urethra/surgery”[MeSH Terms]) AND Humans[Mesh]”. We also used term “human” as limiting term to exclude every study that was not conducted on human subjects.

### Study selection and data extraction

A single reviewer screened the articles based on the titles, abstracts, and full text. Then, the data on author, publication year, details of studies subjects, details of studies intervention, and results are extracted.

### Risk of bias sssessment

Another single reviewer assessed the risk of bias using the Cochrane Risk of Bias Tool, which include random sequence generation, allocation concealment, blinding of participants and personnel, blinding of outcome assessment, incomplete outcome data, and selective reporting. Every domain was judged with 3 levels (low risk, unclear risk, and high risk).

### Analysis and concluding the review

We evaluated the study using appraisal worksheet for randomized clinical trial from Oxford University Center for Evidence-Based Medicine to stratify the risk of bias ^6^. Using Revman 5.3 software, recurrence number from all selected studies were analyzed. Data were analyzed using forest plots with calculation of random effects. It was also done using Revman 5.3 to show relative risk/risk ratio for recurrence rate variable dan p-value. We summarized the conclusion of each study at the table along with its appraisal. We decided to use random effects model since the small amount of study and the difference between them.

## Results

### Literature search

**Figure 1.**
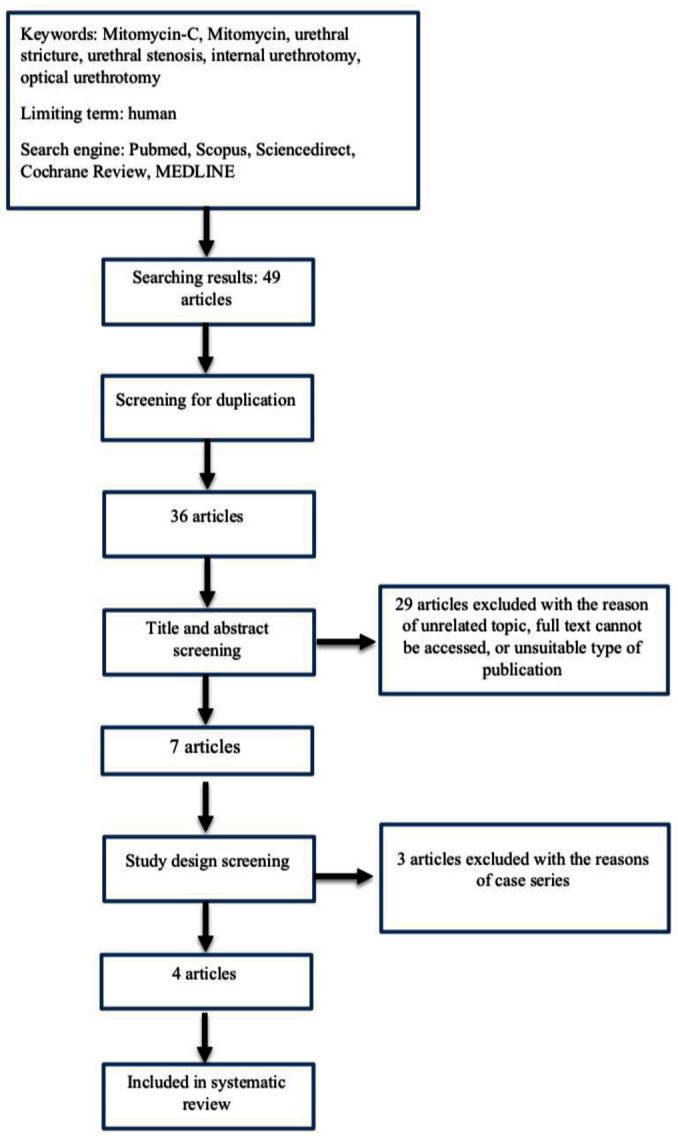
Study flow diagram.

Searching process (searching strategy showed in Figure 1) by using five databases found 49 study articles. There were 13 articles eliminated after screening for duplication. The remained 36 articles were reduced to seven articles after title and abstract screening, leaving seven full text articles to be reviewed. Based on study design, we eliminated three articles, leaving four articles to be summarized in systematic review and meta-analysis.

### Study characteristics and quality assessment

Four selected studies were conducted in 2007, 2015, 2016, and 2019 ^2, 4, 7, 8^. All studies evaluated the effectivity of mitomycin-C given after internal urethrotomy for anterior urethral stricture. From these selected articles, three evaluated the usage of submucosal injection of mitomycin-C for anterior urethral stricture after urethrotomy. How mitomycin-C was injected differed in every study. Mazdak *et al.* ^4^ study used 0,1 mg of Mitomycin-C in 2 ml of distilled water injected in four quadrants, Ali *et al.* ^2^ study used 0,1% Mitomycin-C injected in three quadrants, and Islam *et al.* ^7^ study used 0,1 mg in 2 ml of distilled water in two quadrants. Moradi *et al.* ^8^ study evaluated intraluminal injection of Mitomycin-C in hydrogel base, consisting of 0.8 mg Mitomycin-C with 1cc water and propylene glycol to PF-127 poloxamer. The hydrogel base was injected through a small feeding tube to reach the site of stricture. All studies applied mitomycin-C after internal urethrotomy procedure and were conducted in populations with different age means. Each of studies’ quality was assessed using guide from Oxford University Center for Evidence-Based Medicine; this is explained in Table 1 and Table 2.

**Table 1.** Characteristics of subjects in included studies.

Studies	Average age (year)		Pre-intervention stricture site		Procedure of internal urethrotomy	Clinical feature				Cause of injury	
	MMC group	Control group	MMC Group	Control group		Urinary retention	Bladder Outlet Obstruction	Azotemia	Urosepsis	Mitomycin-C Group	Control group
Moradi *et al*., 2016 ^8^	54.55 ± 21.25	53.75 ± 24.75	Anterior urethral stricture		Trans-urethral incision at 12 o’clock via cold knife urethrotomy	N/A				N/A	
Ali *et al*., 2015 ^2^	37.31 ± 10.1	40.1 ± 11.4	Bulbar urethra: 84.6% Penile urethra: 15.4%	Bulbar urethra: 78.1% Penile urethra: 21.9%	Internal Optic urethrotomy	Mitomycin-C group				Road traffic: 59% Iatrogenic: 30.8% Straddle: 9% Infective: 1.3%	Road traffic: 71.2% Iatrogenic: 21.9% Straddle: 6.8% Infective: -
						70.5%	16.6%	10.2%	2.5%		
						Control group					
						57.5%	31.5%	9.5%	1.3%		
Mazdak *et al*., 2007 ^4^	29.8 (15–70)	29.2 (11–66)	Anterior urethral stricture		Trans- urethral incision at 12 o’clock via cold knife urethrotomy	N/A				Straddle injury or other blunt perineal trauma	
Islam *et al*., 2019 ^7^	49.43 ± 8.10	48.98±7.20	Anterior urethral stricture		Internal Optic Urethrotomy	N/A				Idiopathic, lichen sclerosus, urethritis, or unknown	

[i] MMC, mitomycin C.

**Table 2.** Summary and appraisal of the selected articles.

Studies	LoE	Sample size	Methods of mitomycin- C application	Timing of mitomycin- C application	Follow up end- point	Validity					Importance	Applicability				
						Randomized allocation	Similarity of group	Equal Treatment	Minimal Loss to Follow- up	Blinding		Relevance	Feasibility	Benefit Overweight the Harm
Moradi *et al.*, 2016 ^8^	1b	40	Intraluminal injection of 0.8 mg mitomycin-C + propylene glycol through indwelling catheter	After Internal Urethrotomy	12 months	Not stated	Yes	Yes	Yes	Not stated	RR = 0.20 ARR = 0.40 RRR = 0.80 NNT = 2.5	Unsure	Yes	Yes
Ali *et al.*, 2015 ^2^	1b	180	Submucosal injection of 0.1% mitomycin- C at three quadrants (1, 11, & 12 o’clock position) using TLA needle	After Internal Urethrotomy	18 months	Yes	No	Yes	Yes	Not stated	RR = 0.38 ARR = 0.23 RRR = 0.62 NNT = 4.35	Unsure	Yes	Yes
Mazdak *et al.*, 2007 ^4^	1b	40	Submucosal injection of 0.1 mg mitomycin- C in four quadrants (1,5,7, & 11 o’clock position) using 22- Gauze cystoscopic needle	Before Internal Urethrotomy	6 months	Not stated	Yes	Yes	Yes	Not stated	RR = 0.20 ARR = 0.40 RRR = 0.80 NNT = 2.5	Unsure	Yes	Yes
Islam *et al.*, 2019 ^7^	1b	80	Submucosal injection of 0.1 mg Mitomycin- C in two quadrants (11 & 1 o’clock) using 21-gauge cystoscopic needle	After internal Urethrotomy	6 months	Yes	Yes	Yes	Yes	Not stated	RR = 0.62 ARR = 0.25 RRR = 0.38 NNT = 4	Unsure	Yes	Yes

[i] LoE, level of evidence; RR relative risk; ARR, absolute risk reduction; RRR, relative risk reduction; NNT, number needed to treat.

### Outcome measures

We included the studies in which recurrence was defined by a patient having obstructive symptoms, obvious stricture at retrograde urethrography, or uroflowmetry with maximum flow rate less than 12 mL/s, and stricture was measured using retrograde urethrogram or ultrasonography of the urethra. The outcome that measured was recurrence rate (percentage).

### Risk of Bias

**Figure 2.**
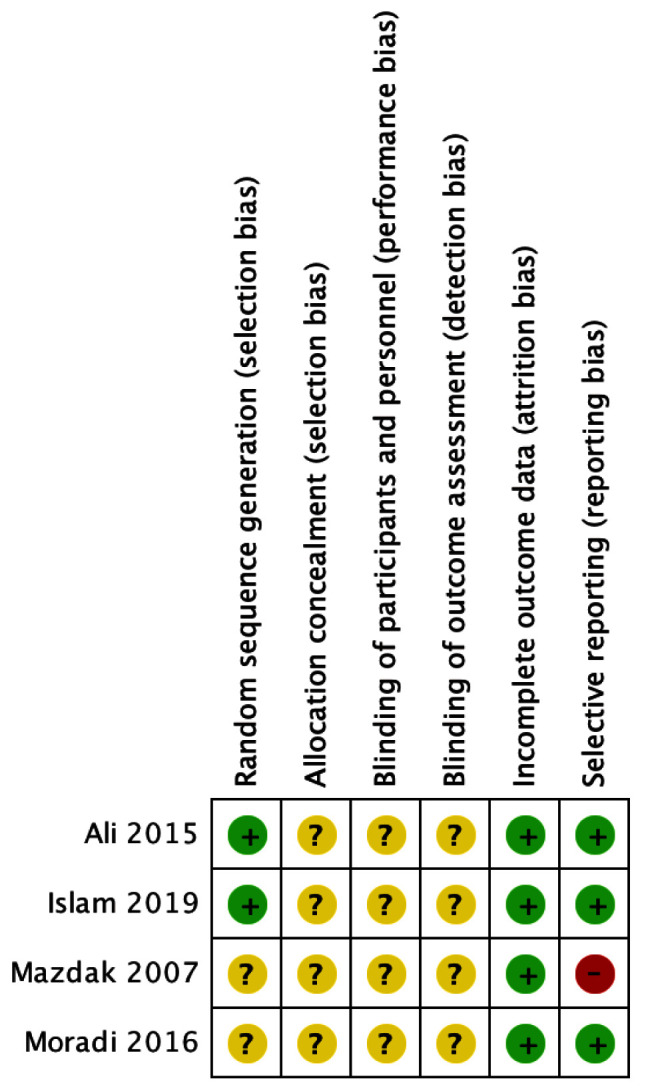
Risk of Bias Summary.

The summary of the risk bias assessment can be found in Figure 2. All of the studies stated that they have undergone randomization. But only Ali *et al*. and Islam *et al*. mentioned the randomization method. A common problem with all the studies included in this review is that there was no clear concealment and blinding statement. All of the studies are completed and have relatively small loss to follow-up rates. Mazdak *et al*. didn’t mentioned recurrence criteria resulting high risk in selective reporting, but other studies mentioned ^2, 4, 7, 8^.

### Results and heterogeneity of the studies

**Figure 3.**
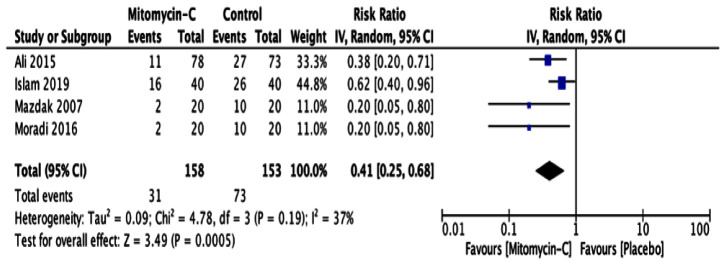
Forest plot for recurrence rate. Test for heterogeneity Tau-square = 0.09; chi-square = 4.78; df = 3; p-value = 0.19; I-squared = 37%.

All selected articles stated that Mitomycin-C had a significant effect on preventing or delaying urethral stricture recurrence post internal urethrotomy. All studies reported that the time-based recurrence rates in the two groups differed, where lower recurrence rates were found in the group given Mitomycin-C ^2, 4, 7, 8^. From study characteristic that is quite different and small number of studies, we choose to use random-effects model in forest plot showed in Figure 3. This forest plot suggests that there were significant differences between cases and control group. It showed from risk ratio is 0.41 with 95% confidence interval of 0.25 until 0.68, with p value <0.05. As for side effects are reported minimal and insignificant ^2, 8^.

## Discussion

All the studies included in this review treated the two groups equally and had relatively small loss-to-follow-up rates. A common problem with all the studies included in this review is that there was no clear blinding statement. Mazdak *et al.* ^4^ and Moradi *et al.* ^8^ study stated that their studies are randomized. But, the randomization procedure was not stated in the study method. On the other hand, although Ali *et al*. ^2^ had randomized its subjects, age characteristics in the two groups were significantly different. Mazdak *et al.* ^4^ also didn’t mentioned recurrence criteria, which could result in high risk bias. There was also a relevant study by Azzawi *et al.* ^9^ that matches our inclusion criteria. But we are unable to access the full text, so we decide to exclude it. As for heterogeneity, from the calculation might not be important difference (I ^2^=27%, p = 0.19). But the small number of studies, that calculation may not work well. From the studies we could notice the difference in age and methods in administering Mitomycin-C, those difference might add up the heterogeneity.

All studies support the use of mitomycin-C to prevent or delay anterior urethral stricture after internal urethrotomy, which in this review defined by a patient having obstructive symptoms, obvious stricture at retrograde urethrography, or uroflowmetry with maximum flow rate less than 12 mL/s, and every stricture in this review are all primary stricture.. This was confirmed by a less rate of recurrence rate in Mitomycin-C patients ^2, 4, 8^; we found that those who had Mitomycin-C administered had lower incidence of recurrence during one year and 18 months of follow up (RR = 0.32, P < 0.001). This was also confirmed by a series of cases by Farrell *et al.* ^10^, Farrell *et al.* ^11^, and Sourial *et al.* ^12^ Mazdak *et al.* ^4^ injected mitomycin-C into the urethral submucosa and reported that patients with mitomycin-C injection had lower rates of stricture recurrence. Opposing this study, some researchers proposed that submucosal injection could increase the complication rate and reduce the duration of the effective dose within the tissue, which yielded a scientific discussion ^13^. Ayyildiz *et al.* ^14^ assessed the efficacy of Mitomycin-C for preventing urethral scar by applying the agent topically to the traumatized region in rats. They concluded that mitomycin-C applied locally reduced fibrosis significantly in a dose-independent manner.

Although all studies support the use of Mitomycin-C to prevent or delay post urethrotomy urethral stricture and the side effects reported in the studies reviewed are minimal, in Ali *et al.* ^2^ and Moradi *et al.* ^8^ are insignificant, but Mazdak *et al.* ^4^ and Islam *et al.* ^7^ didn’t asses any side effects, the results of this review need to be followed up with caution. The limitation of this study can be seen from only a few studies that discuss this topic. Some of the existing studies are not enough to be generalized to a wider population, given that selected studies were carried out in Iran and Pakistan ^2, 4, 7, 8^. Therefore their application needs to be carried out wisely and cautiously. Research related to this in the future can still be done with different populations.

Due to short period of follow up time in all studies, some authors ^2, 4^ concluded that the study of Mitomycin-C needed firm results regarding long term success. Mazdak *et al.* ^4^ added that stricture may recur within two years after internal urethrotomy.

## Conclusion

Mitomycin-C could be used as a potential additional treatment for anterior urethral strictures after internal urethrotomy. However, further studies are required to investigate the safety and efficacy of this method for treating anterior urethral strictures, as only a limited number of studies presently exist.

## Data availability

### Underlying data

All data underlying the results are available as part of the article and no additional source data are required.

### Reporting guidelines

Open Science Framework: PRISMA checklist for ‘Efficacy of mitomycin-C on anterior urethral stricture after internal urethrotomy: A systematic review and meta-analysis’. https://doi.org/10.17605/OSF.IO/APU9B ^15^.

The updated PRISMA checklist is available under the terms of the Creative Commons Zero "No rights reserved" data waiver (CC0 1.0 Public domain dedication).
