# Supplementary material for: Efficacy of mitomycin-C on anterior urethral stricture after internal urethrotomy: A systematic review and meta-analysis
Source: F1000Res. 2020 Aug 10;8:1390. Originally published 2019 Aug 8. [Version 3] doi: 10.12688/f1000research.19704.3 (PMC9843087; doi:10.12688/f1000research.19704.3)
Supplement: Peer review history

# Peer review: Reviewer response for version 3

**DOI:** 10.5256/f1000research.28494.r157926

**Published:** 2023-01-16

**Authors:** Muhammet İrfan Dönmez

**Affiliations:** 1 Istanbul University, Istanbul, Turkey

In this study, the authors aimed to investigate the efficacy of Mitomycin-C in adult patients with anterior urethral stricture after internal urethrotomy. Three studies involving 311 patients were included and a systematic review was conducted. However, I do have some questions and concerns outlined below about their study.

1. According to abstract, three studies involving 311 patients were included in this review. However, flowchart indicates that four articles were included. Please clarify.
2. The search was done in March 15th 2020. If there are other current studies from the last two years, I believe that they should be updated, as well.
3. The review mentioned different usage and dosage of submucosal injection of mitomycin-C in four studies. Is it possible to give an opinion to the reader about which application method is more ideal? The difference and heterogeneity in age and methods in administering Mitomycin-C may cause misinterpretation of the results.
4. *We included the studies in which ...... recurrence was defined .... uroflowmetry with maximum flow rate less than 12 mL/s*. What are the references that the mentioned studies use to determine this threshold value?
5. There was no assessment of side effects in two of the studies. In addition, the lack of clear concealment and blinding statement in all four studies included in this systematic review increases the risk of bias. Further, the time to recurrence cannot be clearly evaluated due to short follow-up period. This raises doubts about the reliability of the study outcomes. As a result, the main limitation of this systematic review is that the results cannot be generalized to a wider population as it only included a few studies. Based on this, giving the message in the conclusion that Mitomycin-C has potential for efficacy in the treatment of anterior urethral stricture after internal urethrotomy may mislead the reader.
6. There several errors of grammar and syntax that need attention.

Are the rationale for, and objectives of, the Systematic Review clearly stated?

Yes

Is the statistical analysis and its interpretation appropriate?

Yes

Are sufficient details of the methods and analysis provided to allow replication by others?

Yes

Are the conclusions drawn adequately supported by the results presented in the review?

Yes

Reviewer Expertise:

urethral stricture

I confirm that I have read this submission and believe that I have an appropriate level of expertise to confirm that it is of an acceptable scientific standard, however I have significant reservations, as outlined above.

# Peer review: Reviewer response for version 3

**DOI:** 10.5256/f1000research.28494.r69072

**Published:** 2020-08-18

**Authors:** Laetitia M. O. de Kort

**Affiliations:** 1 University Medical Centre Utrecht, Utrecht, The Netherlands

I am satisfied with the adjustments.

Are the rationale for, and objectives of, the Systematic Review clearly stated?

Yes

Is the statistical analysis and its interpretation appropriate?

Yes

Are sufficient details of the methods and analysis provided to allow replication by others?

Partly

Are the conclusions drawn adequately supported by the results presented in the review?

Partly

Reviewer Expertise:

NA

I confirm that I have read this submission and believe that I have an appropriate level of expertise to confirm that it is of an acceptable scientific standard.

# Peer review: Reviewer response for version 2

**DOI:** 10.5256/f1000research.26186.r64256

**Published:** 2020-06-29

**Authors:** Farhad Shokraneh

**Affiliations:** 1 King's College London, London, UK

I thank the authors for applying a few of my suggestions:

- Running an update search and adding one of my suggested studies;
- Changing the Fixed-Effect Model to Random-Effects Model (Effects not Effect);
- Balancing their conclusion.

The manuscript can benefit from a native language editor.

 Unfortunately, the majority of the comments and concerns are still standing: revising search strategy involving a search expert, restructuring inclusion and exclusion criteria, reporting/listing/analysing side effects as outcomes, adding new text headings in Methods for screening, data extraction, assessment of risk of bias, and explaining the processes that require involving at least two reviewer, following PRISMA checklist items, discussing the results of heterogeneity test, removing first four paragraphs from discussion or moving them to introduction, and assessing, including or citing Azzawi's study or mentioning the reason for its exclusion.

 The authors have removed EBSCOhost and Ovid rather than mentioning the names of the listed of searched databases via these search interfaces.

Are the rationale for, and objectives of, the Systematic Review clearly stated?

Yes

Is the statistical analysis and its interpretation appropriate?

Partly

Are sufficient details of the methods and analysis provided to allow replication by others?

No

Are the conclusions drawn adequately supported by the results presented in the review?

No

Reviewer Expertise:

Evidence Synthesis, Systematic Review, Information Retrieval, Automation of Systematic Reviews, Living Evidence, Scoping Review, Cochrane Review, Database, Clinical Practice Guideline, Overview, Rapid Review, Randomized Controlled Trial, Open Data, Medical Journalism, Outcome, Intervention, Cochrane.

I confirm that I have read this submission and believe that I have an appropriate level of expertise to confirm that it is of an acceptable scientific standard, however I have significant reservations, as outlined above.

# Response

**Published:** 2020-07-24

**Authors:** Andy Andy

**Affiliations:** Indonesia University, Urology Department, Indonesia

Thank you for your feedback, I really appreciate it. As for the method section and other feedbacks we’ll be revising it. As for the journal database, we have changed from OVID and EBSCOHost with MEDLINE.

# Peer review: Reviewer response for version 2

**DOI:** 10.5256/f1000research.26186.r64255

**Published:** 2020-06-18

**Authors:** Laetitia M. O. de Kort

**Affiliations:** 1 University Medical Centre Utrecht, Utrecht, The Netherlands

My comments:

- The length of the stricture is important indeed and should not be left out.
- Still the definition of stricture recurrence is not mentioned in the discussion.
- Side effects and adverse events are still not mentioned.

Are the rationale for, and objectives of, the Systematic Review clearly stated?

Yes

Is the statistical analysis and its interpretation appropriate?

Yes

Are sufficient details of the methods and analysis provided to allow replication by others?

Partly

Are the conclusions drawn adequately supported by the results presented in the review?

Partly

Reviewer Expertise:

NA

I confirm that I have read this submission and believe that I have an appropriate level of expertise to confirm that it is of an acceptable scientific standard, however I have significant reservations, as outlined above.

# Response

**Published:** 2020-07-24

**Authors:** Andy Andy

**Affiliations:** Indonesia University, Urology Department, Indonesia

Thank you for the feedback again. We really appreciate it. For the length of stricture after critical reading over the references, only Mazdak et al compare the length of the stricture. Meanwhile, others did not compare it even with the newest reference. The definition of stricture recurrence has been mentioned in outcome measures inside the result section, but I will add it to the discussion. As for side effects and adverse events that have been mentioned in the seventh paragraph and the references didn't mention specific side effects nor adverse events, they only mention insignificant and minimal.

# Peer review: Reviewer response for version 1

**DOI:** 10.5256/f1000research.21610.r59109

**Published:** 2020-02-17

**Authors:** Farhad Shokraneh

**Affiliations:** 1 King's College London, London, UK

The authors have conducted the first systematic review to answer this research question. It is a valuable effort.

**Abstract**

1. EBSCOhost and Ovid are not databases. They are search interfaces or search engines for other databases. Please add the name of the databases that you search using these two tools.
2. Please add the exact search date (dd/mm/yyyy).
3. Please state the reason for not using Cochrane Risk of Bias tool for assessing risk of bias of RCTs.
4. Please add rationale for using fixed effect model.
5. The conclusion is so strong. With limited evidence, it is not easy to say that MMC has safety, efficacy, or long-term efficacy.

**Methods**

1. The search date is older than a year (17 months old). Please update the search. I spent a few minutes and identified following recent studies:
  - Azzawi (2018) ^1^
  - Islam *et al. *(2019) ^2^
2. EBSCOhost and Ovid are not databases. They are search interfaces or search engines for other databases. Please add the name of the databases that you search using these two tools.
3. The search strategy reported for PubMed needs serious attention by a search expert such as a librarian or information specialist and through using PRESS checklist for peer-reviewing search strategies. If authors have no access to such person they can post a task on taskexchange.cochrane.org and request a volunteer to help them in exchange to offering Acknowledgement or Authorship, depending on their contribution.
4. When the number of results is little, usually the authors should seek other sources such as references of included studies, citations to relevant studies, contacting the authors of relevant studies, etc. Depending on amount of time and resources available for the team, I request them to use other methods to make sure no studies are missing.
5. Please structure Inclusion and Exclusion Criteria to subheadings: Patients, Intervention, Control/Comparison, and Primary and Secondary Outcomes.
6. I wondered why side effects of interventions have not been listed as collected outcome data.
7. Please add separate headings for Screening, Data Extraction, Assessment of Risk of Bias, and explain their processes.
8. Please follow the PRISMA reporting guideline items one by one.
9. Please state the reason for not using Cochrane Risk of Bias tool for assessing risk of bias of RCTs while it is available within RevMan.
10. Please share your RevMan file as Appendix in Open Science Framework so that the readers could reproduce your analysis.
11. Please add rationale for using fixed effect model.

**Results**

1. Please follow the original PRISMA flow diagram in which duplication is before screening.
2. I2 is not the only way to notice heterogeneity. Actually, I2 may not work well with small sample size and low number of studies. The other ways is to look at the heterogeneity in study details such as characteristics of population (age – compare Age in Moradi with age in Mazdak –, sex, etc.), interventions (method of administration or dosage), and outcomes (methods of measurement). Also N/As in the table are missing details that you may get contacting the authors and they may contribute to heterogeneity. Based on what I see in your description, the method of administration is different among studies plus age of the patients varies across studies.

**Discussion**

1. The first four paragraph of the discussion are not discussing the results and they seem to be relevant to Background.
2. Impact of small sample size, heterogeneity of included studies, adverse effects, subjectivity of outcome measure, not using placebo for control group on interpreting these results are some of the items that could have been discussed here. I also refer the authors to a commentary written by Ng and Chan on Mazdak *et al*. as the first RCT ^3^.

I thank the authors for listing some of the limitations this reviews. I think a new version of this review should be submitted, before being approved.

Are the rationale for, and objectives of, the Systematic Review clearly stated?

Yes

Is the statistical analysis and its interpretation appropriate?

Partly

Are sufficient details of the methods and analysis provided to allow replication by others?

No

Are the conclusions drawn adequately supported by the results presented in the review?

No

Reviewer Expertise:

Evidence Synthesis, Systematic Review, Information Retireval, Automation of Systematic Reviews, Living Evidence, Scoping Review, Cochrane Review, Database, Clinical Practice Guideline, Overview, Rapid Review, Randomized Controlled Trial, Open Data, Medical Journalism, Outcome, Intervention, Cochrane.

I confirm that I have read this submission and believe that I have an appropriate level of expertise to state that I do not consider it to be of an acceptable scientific standard, for reasons outlined above.

## References

[1] : [15] The role of mitomycin C intralesional injection during visual internal urethrotomy in urethral stricture recurrence. *Arab Journal of Urology* .2018;16(sup1) : 10.1016/j.aju.2018.10.062 S8-S9 10.1016/j.aju.2018.10.062 29713532

[2] : Role of Mitomycin C in Anterior Urethral Stricture Recurrence after Internal Urethrotomy. *Pakistan Journal of Medical and Health Sciences* .2019;13(2) :497-499

[3] : Re: Hamid Mazdak, Iraj Meshki and Fatemeh Ghassami. Effect of Mitomycin C on Anterior Urethral Stricture Recurrence after Internal Urethrotomy. Eur Urol 2007;51:1089–92. *European Urology* .2007;52(3) : 10.1016/j.eururo.2007.05.014 10.1016/j.eururo.2007.05.014 17157434

# Response

**Published:** 2020-04-27

**Authors:** Andy Andy

**Affiliations:** Indonesia University, Urology Department, Indonesia

Thank you for the feedback, we have revised according to most of your comments. Update also has been implemented. As for contacting the authors for N/A in tables, we have tried contacting the authors but there is no answer until now.

# Peer review: Reviewer response for version 1

**DOI:** 10.5256/f1000research.21610.r59108

**Published:** 2020-02-03

**Authors:** Laetitia M. O. de Kort

**Affiliations:** 1 University Medical Centre Utrecht, Utrecht, The Netherlands

The subject of this review is interesting and relevant to clinical practice. The review is well written. I have some comments:

- The literature search was until September 2018. Why is an update missing?
- Complications and side effects of the MMC injections were not mentioned.
- There was no clear definition of recurrence of the stricture. As this is a major subject of dispute in urethral surgery, this issue should be addressed, at least in the discussion.
- The length of the stricture was not mentioned.
- Were these all primary strictures? Should be addressed.
- The first publication was in 2007. Yet, MMC injection is not implemented in clinical practice for prevention of urethral stricture. It should be discussed why this is the case.

Are the rationale for, and objectives of, the Systematic Review clearly stated?

Yes

Is the statistical analysis and its interpretation appropriate?

Yes

Are sufficient details of the methods and analysis provided to allow replication by others?

Partly

Are the conclusions drawn adequately supported by the results presented in the review?

Partly

Reviewer Expertise:

NA

I confirm that I have read this submission and believe that I have an appropriate level of expertise to confirm that it is of an acceptable scientific standard, however I have significant reservations, as outlined above.

# Response

**Published:** 2020-04-02

**Authors:** Andy Andy

**Affiliations:** Indonesia University, Urology Department, Indonesia

Thank you for your review. We have revised our publication according to your comments and we will upload it as soon as possible. But some comments I would like to answer them

- The literature search was until September 2018. Why is an update missing?

We have searched with our search strategy and there are no updates until September 2018. 

- The first publication was in 2007. Yet, MMC injection is not implemented in clinical practice for prevention of urethral stricture. It should be discussed why this is the case. It has been stated clearly that the evidence for MMC injections are not enough for it to be implemented in clinical practice. Therefore, we require further research.

We are open to any suggestion for improving this study, thank you
